# Introductory Address

**Published:** 1869-11

**Authors:** H. W. Boyd

**Affiliations:** Chicago


### CHICAGO MEDICAL EXAMINER.

N. S. DAVIS, M.D., Editor.

VOL. X. NOVEMBER, 1869. NO. 10.

### Original Contributions.

ARTICLE XXXIX.

### INTRODUCTORY ADDRESS.

By H. W. BOYD, M.D., Chicago.

Ladies and Gentlemen:—I think I can read in your faces, as I see them before me, the story of a pleasant summer, whose brightness and bloom have given to you vigor and strength for the days which are before us. Many of you are called from far distant homes—some from the farm, and others from their merchandise; and we welcome you to this, the inauguration of the eleventh annual course of instruction in the Chicago Medical College. Since the last medical class passed from the guidance of the Faculty, important changes have been effected in connection with this institution; all of which are hoped to be but milestones of progress in her history. The Chicago Medical College has become the Medical Department of the North-Western University, a relation which it is believed will prove to the mutual advantage of all concerned; and having always entertained liberal views in regard to education, she has extended her liberality, so as to admit to her classes such females as may desire to pursue the study of medicine. This is an age of progress in almost every department of science or art. Philosophy is carrying her researches everywhere, mingling her lessons of wisdom with her lofty speculations. Science is continually unfolding new glories, and adding new conquests to her long lists of triumphs, and at the same time is gradually, but surely, wending her way to the more perfect appreciation of the masses. And not only are the arts and sciences apparently advancing, but public education, societies for the promotion of knowledge, the spirit of invention and discovery, manufactures, and facilities for rapid communication and familiar intercourse, navigation, and all things useful and beautiful excite the attention of the world, and seem to prosper by the labors for their advancement. Large cities are built up in a day, and institutions of learning multiply rapidly. Only one hundred years ago, the first medical school was established in America; but to-day over forty are represented in our various cities, and a much greater ratio of increase can be ascribed to other schools and colleges. But a few years since, and the stage coach and the emigrant wagon were the principal means of transit from place to place; but today, long lines of railroads thread every state and county in the nation, uniting with their iron bands the Atlantic with the Pacific. But by the side of this progress there is a spirit of rash innovation, a seeming desire to blot out the past, the evident possession of wild, bewildering ideas, on almost every subject; a daring, restless inquisitiveness; a discontent; a disposition to hasten all events; to accomplish much in a very short time, and a reluctance to submit to that course of things which is favorable to calm meditation, and clear, steady thought. The evils of such a state of things are manifest in a thousand different forms, but nowhere more clearly than in the tendency to prevent thorough education. Our American people are too much of a money-loving people, they are too busy to think clearly and steadily, and investigate thoroughly and fully, and the result is, we have a paradise for quacks in education. Everything must be done in a day. Our schools and colleges, partaking of the spirit of our people, attempt to do too much in too short a space of time. An education cannot be procured in a month nor a year, but only by the slow process of *years* of labor. And to attain to *eminence* in any department of science or art, entails upon its votaries a degree of enthusiasm and devotion marked by self-denial and indomitable perseverance, and the end is obtained only in proportion as we have labored to obtain it. It is not only during your pupilage that you are expected to study. Those of you who have had preceptors, have just been receiving the initiatory instruction necessary to prepare you for the lectures, so that you can derive the best advantage from the knowledge communicated to you; and what you learn while you are here, in addition to that which you bring with you, will prepare your minds for the reception of more, and thus, as the bounds of your knowledge widen, as the field of your mental vision is extended, new beauties will appear on the distant horizon, and your desires will increase in proportion to your capacities for enjoyment, and the ends you have in view in seeking knowledge. And thus it must be through life. You should always be students, even after you have left the walls of this College, for then you have just begun to climb the hill of science, which is one of steep ascent. There is no railroad communication to its summit, by which passengers can be delivered at the other end of the track at so much a head; it cannot be reached by the aid of the steam car, and amid the confused rattle of machinery; neither can the lightning carry the rich products of those bright fields to the vale below upon magnetic wires; a tariff of prohibition hems its riches in, and it is only by the slow process of days and nights of toil and labor that the end can be obtained. There is but the one course, and no clipping off of corners or taking shorter roads. Every curve, corner, and stone of the pathway must be examined by him who would succeed. And although a process of toil, yet around the hopeful and persevering traveler to truth, thousands of joys are clustered. How his heart leaps at every step in the progress gained, and with what ecstacy he stands upon some jutting rock, which from below seemed an impassable barrier to his higher ascent, and at every resting place in his pilgrimage he discovers the sculptured monuments of those who have gone before him. Here are the trees and shrubs which they have planted, still blooming, and the rich fruits of their labors hang melting to his taste. All along up the richest ores shine from the hillside; an emerald here and there lies embedded, and the pure diamond, like the radiance of the evening star, sparkles upon the vision. Upon the very thorns which pierce his feet, roses bloom, and from every jutting rock, plants shoot out redolent with the perfumes of Eden. Enthusiastic and persevering industry bring an indispensible requisite to success. It may not prove an unprofitable waste of time, to inquire at the starting point of the road which opens so invitingly its broad entrance before us, how we can best employ the future, so as to derive the greatest amount of good.

The Faculty of this College have adopted a course of instruction for which they claim peculiar merits, and its continued and increased success depends largely upon the vim and energy with which you pursue it. You must consider the fact that you are now entering upon the study of the most *noble* and *responsible* of all professions—one that requires men of the strictest honor and integrity, for to physicians are confided the secrets of families, sacred trusts, that are committed to no others. The high and noble motive of doing good to others, of relieving human suffering, and prolonging human life, is the only incentive that ever has, or ever will make the great physician. And if he has no nobler and higher motive than the mere gain that can be made by it, he is unfit to be trusted with the lives of human beings. How easy it is for the physician to control the destiny of his patients. In him they trust, and confide in his knowledge and TRUTH. He decides for them questions of life and death. Happiness or misery, it is his power to give. Because by devoted study, he has learned the mysteries and marvels of this wondrous machine, the human body. He has learned the anatomy of every part and organ, and of the laws which govern and regulate them, and of the dire results which follow their neglect or abrogation. He has also become acquainted with the proper mode of assisting nature to carrying out these laws, so that health can be secured and disease banished. The greater his knowledge the greater his power.

To enable you to contribute, in the highest degree, to the advancement of such holy and philanthropic purposes, it will be necessary that the profession, through whose instrumentality you are to effect so much, should receive your daily and unwearied attention. Now ask yourselves the question, shall you prepare yourselves thoroughly? or shall selfish fear and desire for personal ease prevail, and prevent you from undergoing the labor necessary to qualify yourselves, to assume this fearful responsibility? These are important questions, and upon the answers given them depends the future standing of every medical student in this house. Do not delay to decide whether you will take a noble and elevated rank in your profession, or merely follow it as a trade. Determine now, at the commencement of this term of instruction, and that you will devote all your energies, and all your time, by closest application, to obtain a most perfect and minute knowledge of the elementary branches of your profession, for upon the accuracy and minuteness of your knowledge of these branches—the alphabet of your profession—rests all the superstructure, upon which you are to secure the monument of your future fame. Have it a broad and deep foundation, so that however high your fame may get, it cannot be shaken from its firm foundation. If you are deficient in these elementary branches—the only enduring basis for a permanent reputation—you will never obtain a distinguished position. You must, therefore, see the necessity of the closest application, even at the commencement of your career, for now is the period when these branches are taught. An attempt to gain a knowledge of the beautiful orations, and other compositions of the classic writers, in their classic tongue, without a knowledge of these languages, will fail. So in regard to your profession, the alphabet of which is: Anatomy, Physiology, Chemistry, Materia Medica, and Pathology. And when all these have been learned thoroughly, then you have a good foundation to build up a solid and enduring professional character.

The first great object of education is to discipline the mind, or train the mental faculties so that you have complete command over them. Our earliest knowledge of the human mind is that revelation that the Almighty breathed into man the breath of life, and man became a living soul. The mind is this breath of life, breathed from the lips of the great "*I* AM" into the body already prepared for its reception; and it is that breath of life breathed into no other creature but man, that constitutes him in the image of God. The object of training the mind should be, not only to fulfil her duties well here, but to enable it to stand on high vantage ground, when she leaves this, the cradle of her being. But very few students ever accomplish as much as they expect, or as much as they ought; and one great reason of this is that they fail to obtain this controlling power over the faculties of the mind, because they waste so large a portion of their valuable time. This is the universal experience of students. Every one can look back at his college days, and see the mistakes he made. If the mind has not been trained by previous collegiate or academic studies, you will find the necessity for the closest application all the more urgent in the study of medicine. The EDUCATION of the mind must be your own work. No one can do it but yourself. There is nothing in this world which has not labor and toil as its price. Those islands which so beautifully adorn the Pacific, were reared from the bed of the ocean by the little coral insect, which deposits but one grain of sand at a time, until the whole island is raised above the surface of the waters. Just so with human exertions, the greatest results of the mind are produced by small, but continued efforts.

The mind is endowed with certain faculties or powers, by which it acquires, retains, and digests knowledge; and whenever it has been as highly trained, or developed, as these faculties will permit, and in which it has its greatest power, precision, and readiness of action, then it may be said to be educated. A person may have a good memory, retentive to the minutest fact; he may be a keen observer of things passing around him, or an extensive and diligent reader, and, indeed, may have capacity to acquire and retain knowledge of a rare order; and if this be all, he may still be very far from being thoroughly educated. Many uneducated men are diligent readers, and have good memories, but the end of education is not simply to acquire knowledge. The food we eat, we employ not as an end, but as a means; the end is that the body be strengthened and developed, which is not accomplished by simply eating the food, but only when it has been duly digested and assimilated. So it is with knowledge, which is to the mind what food is to the body. The exercise which the mind has—for instance, in the study of anatomy or chemistry—disciplines certain of its faculties, so that it is prepared for loftier flights, and to achieve new and higher victories. Learn to think on what you study, so that you can have a good judgment. A well-balanced mind is of most essential importance to the success of a physician. Then your minds cannot only investigate, but weigh and balance the opinions and theories of others. Without this you will never know what to distrust, or what opinions to receive. Some of the most laborious men have passed through life without accomplishing anything desirable, and all for a lack of a well-balanced judgment. The last theory with such a man is the true one, however deficient as to proof; the last book is the most wonderful, though it is in *fact* the most worthless; his last visionary speculation, the one bound to make him rich, though it will produce financial emaciation; hence there is a laborious trifling, which unfits the mind for anything valuable. It leads to a wide field, which is a barren waste.

The old maxim "*know thyself*," is worthy of the attention of the student; there is no knowledge to be compared to self-knowledge. The ancients deemed it worthy to be placed over the doors of their temples, so we imagine they thought highly of this kind of knowledge; and when we reflect that these people have left a deeper impress upon the world's history than any other since time began; an age which gave to poetry a *Homer*, to painting an Apelles, to sculpture a Phidias; which had in statesmanship a Pericles, in law a Solon, in oratory a Demosthenes, and in philosophy a Plato, we are more than ever impressed with the importance of "self-knowledge;" without it we are never rightly prepared to estimate our own powers; we may, as most do, suppose we possess some degree of mental power we do not possess, or, at least, that others will not accede to us; or we may possess faculties of which we are not aware—the former is by far the most likely to be true, for men and women are each possessed with a full share of self-conceit. In your studies you should follow some system, a rule of action, or law. In nature, every effect implies a cause, and every phenomenon implies a law. The combination of the elements in the physical world about us occur in conformity with the laws of chemical union; the forms of the crystal kingdom are built up after the most rigid and beautiful laws; the forms of the plant and animal kingdom, in their development, growth, reproduction, and decay, observe appropriate laws; the wonderful harmony which pervades the system of worlds, wheeling in their immensuosities of space is the result of law; and so the mind, in its search after truth, must do so under the guidance of law. Truth does not wander lawlessly through space, and is not met by the chance of some lucky fool, as it is by the philosopher. Method or law is the leading feature of an educated mind. Another essential quality is *subordination:* this is accomplished through the agency of the will; to be able to control each faculty by means of the will is most desirable, and an exceedingly difficult faculty to obtain. Some men seem to have possessed it to a wonderful extent. When the city of Syracuse was captured by the Romans, Archimides was so much engrossed in one of his problems that he was wholly unconscious of the capture of the city, until he was surprised by a Roman soldier. A famous German philosopher was in the City of Jena at the time of its capture, by Ferdinand, under Napoleon, engaged in writing one of his most profound works; and, on going out to meet his publisher, had his first intimation of the capture of the city by being arrested by a French soldier. Sir Isaac Newton is said to have lived for three years only to think. Sometimes as he would arise from his couch of a morning, he would be arrested by some new conception, and would remain sitting for hours in profound thought, totally unconscious of his situation, or of what passed around him.

My design is to impress your minds deeply that an education is exceedingly difficult to obtain, and that it is a work which few can say they have accomplished, in its fullest sense, and that you will never obtain any degree of it without striving after it. Do not let the difficulties and perplexities of the pathway discourage you: it is a poor ideal that can be reached without time and toil, or that it is possible to surpass in the actual. It is much better for us to have an ideal which we can never quite reach, provided it be a just one, and in the line of possible accomplishment, and that every step we take is towards it, and carries us nearer and higher, and that we feel that we have not a moment to linger, not even the loss of a day, nor an hour. Mental training is obtained in proportion to the time and labor spent. The highest degree possible requires an amount of labor terminated only with one's life. Newton was in his 85th year improving his chronology. The mind becomes possessed of knowledge by means of the senses—we see, we hear, we smell, and we taste; these are our five senses, our perceptive faculties, and are the avenues of knowledge to the mind. By means of our senses we become acquainted with the qualities of things about us; one thing is hard and round, another is soft and square; one is sweet, another *sour*, beautiful, or homely—this is the simplest knowledge, and yet it illustrates how we become possessed of knowledge. Now, this knowledge would do us no good unless we could retain it, so we are provided with a faculty called memory; and then, after we have acquired and retained the knowledge, it would do us but little good if we could not call up from our past experiences what we have, for instance, read, or heard, or seen; consequently, we are provided with another set of faculties, by which we are enabled to classify our knowledge in the storehouse of memory, by which facts possessing certain characters, resemblances, analogies, or differences are grouped together; and by this set of faculties, we can bring up from memory any fact, and compare it with another fact. It is by this set of faculties that we classify and discover general truths, which do not appear at first sight, and by which we discover principles and laws, and institute science and philosophy. We cannot perform an act of memory until we have something to remember, nor can we perform an act of comparison until we have acquired and retained knowledge—then we say that the order of these faculties, in their development and use, is memory, retention, and reflection.

The popular saying that "practice makes perfect" is a true one; it is a law, and applies in many ways, either to the physical, mental, or moral nature. We are familiar with it in physiology, where it may be traced in the paralyzed limb, and in the one daily employed in vigorous exercise, or, for instance, in the muscles in the arm of the blacksmith. The same law holds good for the mind; for by constant vigorous exercise, its faculties become strong and well-developed, and by inaction they become weak and incapable of much effort. You are well aware how difficult it is for a beginner to manipulate the keys of a piano, even in the simplest piece of music, and that after assiduous exercise or practice, a celerity is obtained which almost surpasses belief. This is also true of the mind. Make it your first object to fix and hold your attention upon your studies. You may make many efforts at this without success: this is said to have been the secret plan of Demosthenes, who shut himself up in his celebrated dark cave for study; and this will also account for the fact that persons who are unexpectedly deprived of their eyesight will not unfrequently make advances in thought, before unknown to them. *Patience* is a virtue kindred to attention, and one that is miserably lacked by ninty-nine out of every one hundred of the human race: it disposes to quiet, steady efforts, even in the face of difficulties. Sir Isaac Newton said, the difference between his mind and the mind of others consisted solely in his having more patience. There is no department of human effort in which *patience* is more essential than in the study of medicine. It is impossible to learn everything at once; the tree must grow by inches; patient labor and steady effort will bring everything in its time. It is better to learn less, and do it thoroughly, than to hasten impatiently over a subject. This has been compared to an army conquering a country. If you are patient, and conquer everything before you thoroughly, then you will pass on from victory to victory; but if you have left, here and there, a fort or a garrison unconquered, you will have an army hanging in your rear, and your ground will soon need to be reconquered: so it is in the study of your chosen profession. It is said that the ancients had a great recompense for the scarceness of their books, in the thoroughness with which they were compelled to study them; a book had to be all copied with a pen, to be owned; and he who transcribed it would be likely to understand it. It is said that Demosthenes copied Thucydides ten times. Then it is important for you to be thorough, have a clear understanding of all that you go over. Keep in mind the remarks of the gifted Mr. Wirt: "Take it for granted, there can be no real excellence without great labor."

